# Enhancing semantic question similarity in Arabic using hybrid models and knowledge graph alignment

**DOI:** 10.7717/peerj-cs.3988

**Published:** 2026-07-20

**Authors:** Ammar Al-Edhari, Mohsen Kahani

**Affiliations:** 1 Department of Computer Science, Faculty of Education, University of Kufa, Najaf, Iraq; 2 Department of Computer Engineering, Faculty of Engineering, Ferdowsi University of Mashhad, Mashhad, Razavi Khorasan, Iran

**Keywords:** Arabic NLP, Arabic semantic question similarity, Knowledge graph alignment, Knowledge graph embedding

## Abstract

Semantic question similarity (SQS) plays a crucial role in enhancing question answering (QA) systems, particularly for low-resource languages, such as Arabic, which present challenges due to complex morphology, semantic ambiguity, and limited linguistic resources. Despite prior efforts, knowledge graphs (KGs), which are structured networks representing entities and relationships, remain underutilized in improving semantic understanding and reducing ambiguity. Existing semantic similarity approaches have inherent trade-offs: KGs capture deep meaning but lack adaptability; *corpus*-based methods, such as word embeddings, rely on statistical patterns without deep semantic understanding; and deep learning models efficiently perform but lack interpretability. In addressing these limitations, this study proposes a hybrid SQS model that integrates knowledge graph embedding (KGE) with AraBERT embeddings to enrich semantic representation. The model utilizes a bidirectional long short-term memory network with an attention mechanism to predict question similarity, leveraging the strengths of each approach while mitigating their individual shortcomings. Furthermore, we align LughaNet (a variant of Arabic WordNet) with Wikidata using the similarity flooding (SF) algorithm to enhance KG-based semantic representations, creating a comprehensive and large-scale Arabic knowledge graph. Experimental evaluation on an augmented Mawdoo3 dataset demonstrates that our approach achieves an F1-score of 93.88%, outperforming state-of-the-art benchmarks, including GPT-based models. These findings confirm the effectiveness of hybrid models in capturing structured and contextual semantic relationships, advancing Arabic natural language processing (NLP) research. Additionally, this study introduces a large enriched Arabic knowledge graph, providing a valuable linguistic resource that lays the foundation for future advancements in low-resource language processing, with direct applications in automated QA systems, intelligent search, and recommendation engines.

## Introduction

Semantic question similarity (SQS) plays a crucial role in the initial phase of question analysis within a question answering (QA) system. SQS is widely utilized due to its ability to identify and eliminate duplicate inquiries from community QA platforms, such as Quora. The primary goal of SQS is to find forum questions that are semantically similar to a specific new query. SQS helps save time and effort by reducing the number of duplicate inquiries; if a user’s query is similar to previously asked questions, then a prompt response can be expected.

Despite its advantages, retrieving semantically relevant questions remains challenging because users often phrase their queries differently, leading to irrelevant results and increased complexity. This challenge is even greater for Arabic, a low-resource language characterized by diverse dialects, complex morphology, and strong contextual dependence in meaning (Fuad & Al-Yahya, 2022). Several previous studies (Alkaoud, 2025; Daoud, 2022) have attempted to address these challenges; however, progress remains limited due to the lack of suitable semantic resources, resulting in continued reliance on rule-based methods. While semantic similarity evaluation for languages such as English has reached a mature stage—achieving over 80% correlation with human judgments thanks to rich semantic text similarity (STS) datasets—Arabic still suffers from limited data resources, morphological diversity, and dialectal variation (Fuad & Al-Yahya, 2022). Furthermore, the optional use of diacritics in Arabic introduces additional ambiguity, as undiacritized sentences (*e.g*., “درس محمد الدرس”) may convey different meanings such as “Mohammad studied the lesson” or “Mohammad taught the lesson,” depending on context (Alshammari & Elleithy, 2024; Azmi, Alnefaie & Aboalsamh, 2021; Ismail, Shishtawy & Alsammak, 2022).

Several approaches have been explored to improve Arabic SQS. In earlier work, traditional machine learning techniques were adopted, as demonstrated in the study by Sharifi, Hassanpoor & Maduyieh (2019). *Corpus*-based methods were examined in the research of Othman, Faiz & Smaïli (2019a), while Hamza et al. (2022) combined *corpus*-based and deep learning approaches. Additionally, Boudaa, Haddadi & Boudaa (2024b) investigated the use knowledge graphs (KGs) for synonym identification. Although these studies achieved promising results, most relied on a single paradigm—statistical, semantic, or neural—without effectively integrating their complementary strengths.

A recent study (Chandrasekaran & Mago, 2021) suggests that hybrid models—combining knowledge graphs (*e.g*., WordNet), *corpus*-based representations (*e.g*., word embeddings), and deep learning architectures (*e.g*., Convolutional Neural Networks (CNNs) and Bidirectional Long Short-Term Memory Networks (BiLSTMs))—can more effectively capture semantic similarity by mitigating the inherent limitations of individual approaches. However, Arabic SQS research has yet to fully integrate rich KGs relations—such as synonymy and hypernymy—with contextual embeddings (*e.g*., Arabic Bidirectional Encoder Representations from Transformers (AraBERT)) and deep learning models within a unified hybrid framework. Leveraging resources such as Arabic WordNet and Wikidata within such a framework could significantly enhance semantic understanding and improve question similarity detection.

One of the key challenges in utilizing KGs for SQS is that a single KG is often insufficient to capture diverse semantic relationships. This limitation has driven research interest in entity alignment (EA), which aims to merge overlapping or complementary KGs into a unified representation (Zeng et al., 2021). However, KG alignment for low-resource languages has received limited attention (Tang et al., 2024). Existing models often struggle with Arabic KGs due to the lack of processing tools and annotated resources (Ahmed, Al-Aswadi & Noaman, 2022). This situation highlights the pressing need for further research in Arabic KG alignment techniques.

To address these challenges, this study makes two main contributions. First, it enriches Arabic semantic resources by aligning LughaNet (Al-Edhari & Kahani, 2025)—an extended Arabic WordNet comprising 85,991 synsets—with Wikidata using the Similarity Flooding algorithm. Previous studies, such as Boudaa, Haddadi & Boudaa (2024b), primarily relied on the limited Arabic WordNet (≈11,296 synsets) for synonym extraction, which constrained their ability to capture broader semantic relations. In contrast, the proposed alignment expands semantic coverage by incorporating additional relations such as hypernymy and hyponymy, resulting in a richer and more interconnected KGs.

Second, this study introduces a hybrid BiLSTM-Attention model that integrates knowledge graph embedding (KGE) (*e.g*., Node2Vec) with contextual embeddings from AraBERT, effectively combining structured semantic knowledge with contextual representations. This hybrid design addresses the limitations of prior approaches based solely on fastText or AraBERT embeddings, which often capture surface-level similarities without deeper semantic reasoning. By jointly leveraging KG semantics, contextual embeddings, and deep neural modeling within a unified architecture, the proposed framework exploits the complementary strengths of these paradigms to achieve better semantic precision and improved generalization in Arabic SQS.

The remainder of this article is structured as follows: ‘Related Work’ reviews related work on Arabic SQS and KG alignment. ‘Proposed Method’ outlines the proposed methodology, including alignment techniques, embedding strategies, and the hybrid model architecture. ‘Results and Discussion’ presents the result and discussion, while ‘Conclusions’ provides the conclusion and future research directions.

## Related Work

In this section, we discuss previous work in two topics relevant to our research: Arabic SQS and KG alignment.

### Arabic SQS

Early explorations into Arabic SQS predominantly relied on traditional machine learning techniques that emphasized manual feature engineering and selection. In one study, a rich set of morphological, syntactic, semantic, and lexical features was extracted from the Mawdoo3 dataset and evaluated using multiple classifiers, achieving 82.9% accuracy with random forest feature selection (Al-Asa’d et al., 2019). Another study improved the F1-score from 65% to 84% on 1,450 question pairs using WEKA 3.8, highlighting the contribution of lexical features (Daoud, 2019). Subsequent research optimized Support Vector Machine (SVM) performance (F1 ≈ 82.6%) by selecting the top 38 features *via* the SelectKBest algorithm (Sharifi, Hassanpoor & Maduyieh, 2019), while additional work advanced feature engineering by combining topical and rule-based features, reaching 85% F1-score (Daoud, 2022). Collectively, these studies highlight the importance of diverse feature design in improving Arabic SQS but also reveal its limitations in capturing deeper semantic meaning.

*Corpus*-based methods have substantially advanced Arabic semantic similarity research through static and contextual word embeddings. In one study, Word2Vec combined with K-means and cosine similarity was applied to the Yahoo Answers datasets, achieving a Mean Average Precision (MAP)—which measures overall retrieval precision at different recall levels—of 50.36% in English and 41.44% in Arabic, with the gap attributed to Arabic’s morphological complexity (Othman, Faiz & Smaïli, 2019a). Another study enhanced the Mawdoo3 dataset by combining augmented data with AraBERT embeddings, achieving 96.9% accuracy (AlAwawdeh & Abandah, 2021). Additional research demonstrated the strength of transformer-based models, where a BERT-based approach on Task 8 outperformed Siamese and XGBoost, achieving an F1-score of 92.99% without additional feature engineering (Hammad et al., 2021). Collectively, these studies confirm the effectiveness of contextual embeddings in addressing Arabic’s linguistic and semantic complexity.

Deep learning techniques using satic (non-contextualized) embeddings have also contributed to advancing Arabic SQS. In one study, One-Dimensional (1D)-CNN, BiLSTM, and Bidirectional Gated Recurrent Unit (BiGRU) models combined with Word2Vec embeddings were evaluated, achieving 76.9% accuracy on the NURL 2019 Task 8 dataset, where 1D-CNN performed best (Einea & Elnagar, 2019). Further work on community question answering (cQA) explored question retrieval using Siamese long short-term memory (LSTM) models, improving Mean Average Precision (MAP) from 57.39% to 57.99% for English and from 45.13% to 45.40% for Arabic through the addition of an attention mechanism (Othman, Faiz & Smaïli, 2019b; Othman, Faiz & Smaïli, 2020). A comparative study assessed LSTM-FastText, CNN-FastText, and Transformer-AraBERT architectures, reporting 93% accuracy on the PAN dataset and 83% on Mawdoo3 (Aliane & Aliane, 2020), while another study employing FastText with a feedforward neural network achieved an F1-score of 92.8% (Alkaoud, 2025). Collectively, these studies demonstrate that although static embeddings and deep learning architectures have improved SQS performance, they still struggle to capture deeper semantic relationships in Arabic.

The integration of contextualized embeddings with deep learning has significantly advanced Arabic natural language processing (NLP) by enhancing semantic representation. Al-Bataineh et al. (2019) applied Embeddings from Language Models (ELMo) with a trainable LSTM, achieving an F1-score of 93% for Modern Standard Arabic. Fadel, Tuffaha & Al-Ayyoub (2019) extended the NSURL 2019 Task 8 dataset and employed ELMo with an Ordered Neurons Long Short-Term Memory (ON-LSTM) enhanced by self-attention, obtaining F1-scores of 96.5% (public data test) and 94.8% (private data test), demonstrating improved generalization through data augmentation and attention. Al-Theiabat & Al-Sadi (2020) integrated Recurrent Neural Network (RNN), CNN, multi-head attention, and multilingual BERT, achieving an F1-score of 95.9%, while (Hamza et al., 2022) applied ELMo with a Bidirectional Attention BiLSTM, achieving an F1-score of 93.05%.

In domain-specific contexts, Alshammari & AlHumoud (2022) developed the Tawasul Arabic Question Similarity model using BERT–BiLSTM and Hybrid Transfer (HT)-BERT–BiLSTM, achieving an F1-score of 94.45% on a 44,404-pair higher education dataset. Balla, Llorens Salvador & Delany (2022) introduced the cQA-MD dataset covering both standard and colloquial Arabic, attaining a peak F1-score of 66.75% with AraELMo and ON-LSTM, underscoring the difficulty of domain-specific and medical text processing.

Despite the potential of KG methods, their application to Arabic question similarity remains limited. Keltoum et al. (2021) developed an ontology-based QA system for Islamic banking fatwa questions, but the narrative nature of queries limited the use of formal semantic frames. Boudaa, Haddadi & Boudaa (2024b) utilized Wikidata and Arabic WordNet for synonym extraction and entity normalization; however, their approach focused mainly on synonymy, neglecting richer relations such as hypernymy and hyponymy. These limitations emphasize the need for graph-based models that can effectively exploit structured semantic relations for more accurate Arabic question similarity detection.

Chandrasekaran & Mago (2021) suggested that integrating knowledge-based, *corpus*-based, and deep learning approaches can mitigate the limitations of individual techniques. They advocated the development of hybrid models that combine structured resources, such as WordNet and Wikidata, with pre-trained embeddings like BERT and neural architectures (*e.g*., CNNs or LSTMs). By leveraging the strengths of these paradigms, hybrid models are better equipped to capture the linguistic and semantic complexities of Arabic SQS.

### KG alignment

KGs have become essential tools for organizing and representing complex real-world information in the context of artificial intelligence (AI) and big data. They model knowledge as relational triplets—linking entities (nodes) through semantic relationships (edges)—defined by ontologies that specify domain concepts and their interconnections. Well-known examples include DBpedia, Wikidata, WordNet, and YAGO (Peng et al., 2023).

**Figure 1:**
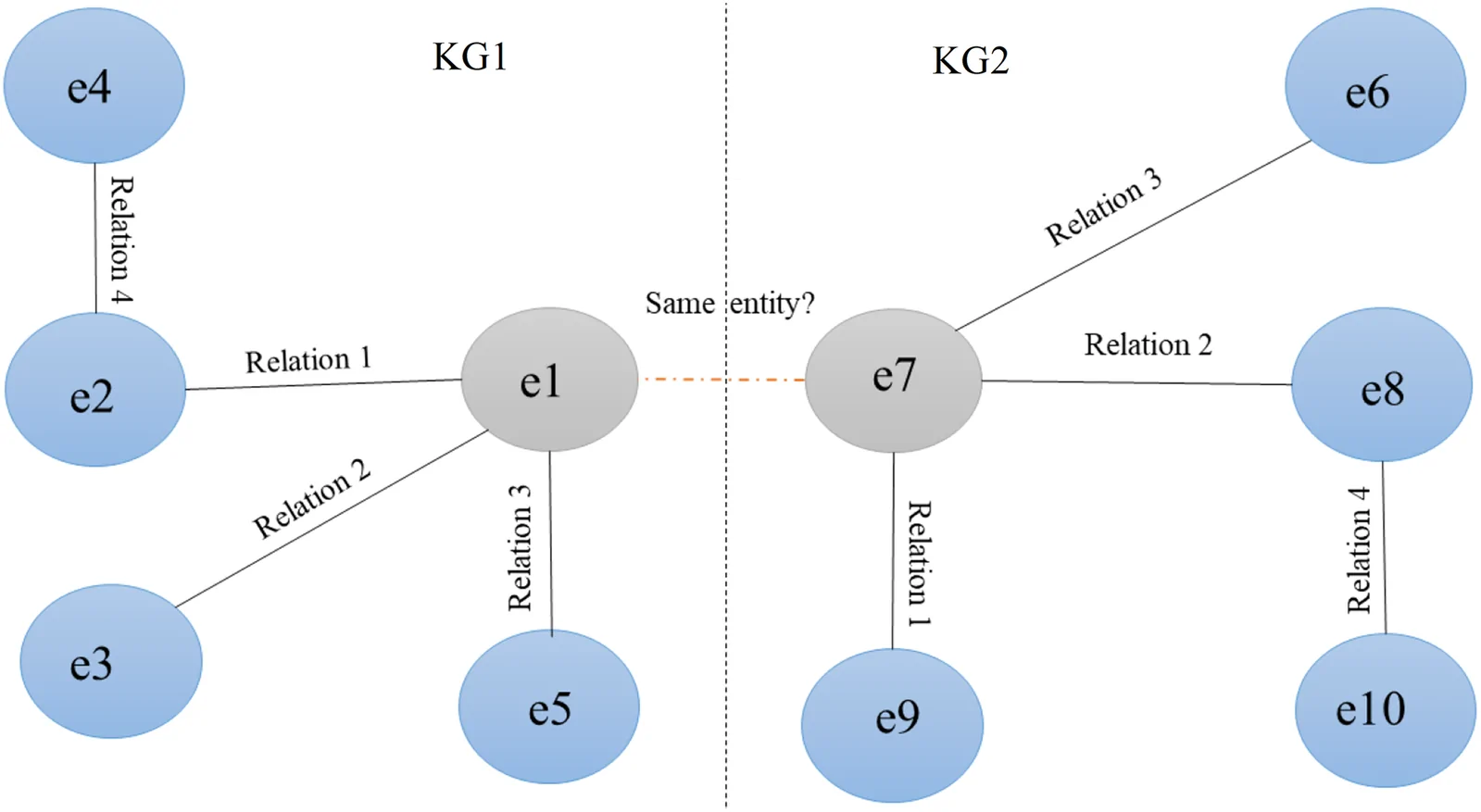
An example of a knowledge graph entity alignment (Lin et al., 2023).

Constructed typically using the Resource Description Framework (RDF), KGs represent knowledge in forms such as “head entity–relationship–tail entity.” For example, the triplet (Milan, country, Italy) expresses that Milan is a city in Italy (Zeng et al., 2021). Such structured representations underpin many AI applications, including recommendation systems (Wang et al., 2021a), semantic similarity (Sousa, Silva & Pesquita, 2024), and question answering (Chen et al., 2021). However, as applications become more diverse, a single KG often fails to provide complete coverage. Consequently, Entity Alignment (EA)—the process of integrating overlapping or complementary KGs—has become a key research area (Zeng et al., 2021). Figure 1 illustrates EA as the matching of equivalent entities across different KGs.

Over the years, progress in monolingual EA has improved the accuracy of matching entities and their relationships in KGs. Marshall, Chen & Madhusudan (2006) introduced the SF algorithm, which focused on propagating similarities across nodes, achieving 91% recall in educational concept maps. This study primarily addressed monolingual data scenarios, providing a foundation for subsequent advancements. Assi & Dhifli (2021) proposed SBIGMat, which utilizes affinity-preserving random walks (APRW) to rank instance pairs by similarity. When SBIGMat was evaluated on OAEI benchmark datasets, including monolingual data, it achieved F1-scores between 88% and 95%, with APRW improving alignment accuracy by approximately 1–4% in noisy and complex scenarios.

Jiang, Liu & Deng (2022) resented FuzzyEA, a model that excelled in cross-lingual and monolingual scenarios, achieving Hits@1 scores of 87.87% on FB15K using techniques such as TransE and fuzzy sets to manage uncertainty, where Hits@1 measures the percentage of correctly aligned entities ranked first among all candidates. Song et al. (2022) proposed BERT and GCN-based Interaction Model for Entity Alignment (BG-INT), which integrates BERT and graph convolutional networks (GCNs) to align entities by combining names, relationships, and attributes. When BG-INT tested was on multilingual and monolingual datasets, it achieved a remarkable HitRatio@1 of 96.5% in monolingual settings while minimizing data redundancy. Wang & Chen (2024) proposed Dense Entity Retrieval for Entity Alignment (DERA) for monolingual and cross-lingual EA, using language models for a unified text conversion. DERA achieved a 98.2% F1-score on D-W-15K (monolingual) and Hits@1 scores of 94.6%, 92.1%, and 94.9% on DBP15K variants (cross-lingual), with slightly higher monolingual performance.

EA tasks face challenges due to the high cost of labeled data, limiting the practicality of supervised methods (Cai, Ma & Jiang, 2024). Suchanek, Abiteboul & Senellart (2011) proposed Probabilistic Alignment of Relations, Instances, and Schema (PARIS), a probabilistic alignment algorithm that can achieve 90% precision without training data. Lacoste-Julien et al. (2013) developed Simple Greedy Matching for Aligning Large Knowledge Bases (SiGMa), a scalable method that can achieve 95% accuracy using iterative alignment with graph structures. Zhang et al. (2023) introduced AutoAlign, which leverages predicate-proximity graphs and embedding, achieving 96.91% Hits@10 on DWY-NB benchmarks. Cai, Ma & Jiang (2024) proposed SLU, an unsupervised model that integrates graph networks and contrastive learning, achieving Hits@1 of 98.4% on the DBP-15K and DWY-100K datasets.

However, low-resource language KGs have received limited attention, and existing models often poorly perform on these KGs (Tang et al., 2024). In the case of Arabic KGs, the scarcity of information processing tools exacerbates this issue, making the integration and fusion of KGs a significant challenge (Ahmed, Al-Aswadi & Noaman, 2022). This situation underscores the need for further research into the alignment of Arabic KGs. Although some progress has been made in constructing Arabic KGs, such as the morpho-semantic KG proposed by Bounhas, Soudani & Slimani (2020) for Arabic information retrieval, these efforts remain limited in scale, domain coverage, and interoperability. Therefore, further research is needed to develop robust Arabic KG alignment techniques capable of integrating heterogeneous resources, such as Arabic WordNet and Wikidata, to support deeper and more comprehensive semantic understanding.

### Proposed method

This section outlines the key steps of our method. The first part focuses on KG alignment and embedding techniques. The second part highlights the architecture of a hybrid model for detecting semantic similarity in Arabic questions.

### KG alignment and embedding techniques

This section focuses on two key components of our method: KG alignment using the SF algorithm and embedding techniques, such as Node2Vec, Graph Attention Networks (GATs), and Graph Sample and Aggregate (GraphSAGE), to represent graph entities and relationships.

#### Alignment KGs using SF

In this study, we align two Arabic knowledge resources: LughaNet and Wikidata. LughaNet consists of 85,991 synsets, making it a notable expansion over the original Arabic WordNet, which contains 11,296 synsets (Cavalli-Sforza et al., 2013). We use the SF algorithm, which operates without training data and is well-suited for Arabic KG alignment, to align these KG resources.

Entities from LughaNet and Wikidata are matched using the SF algorithm, based on lexical and semantic similarity across multiple attributes. Each entity pair is represented by its set of semantic relations (*e.g*., synonyms, hypernyms, and hyponyms), and the cosine similarity between their relation vectors is computed. To ensure reliable alignment, only pairs with similarity scores above an initial threshold of 0.5 are considered matches—a value provisionally selected and subject to further optimization based on experimental results.

The SF algorithm (Melnik, Garcia-Molina & Rahm, 2002) demonstrates its graph-matching and alignment process by constructing a pairwise connectivity graph (PCG) based on the relationships between elements in two models (models A and B). The algorithm computes the final fixpoint values representing the correspondences between elements in the two models through iterative propagation of similarity values, as visualized in the induced propagation graph. This approach, widely applied in schema alignment, knowledge base integration, and data model matching, involves several key steps, which will be further explained:

##### Graph representation

The initial step represents each data model as a directed graph, where nodes symbolize entities, and edges define the relationships between them using triples (source, predicate, and target). This structured representation highlights entity relationships, enabling graph alignment in the algorithm.

##### Similarity propagation graph

Similarity propagation graph is an auxiliary structure derived from two models, A and B, and plays a key role in the algorithm’s fixpoint computation. This approach starts with a PCG, where each node represents a mapping between elements in A and B, and edges encode potential similarity propagation between neighboring pairs. The PCG is expressed as follows:

(1) $$\left( {\left( {x,y} \right);p\left( {x^{\prime},y^{\prime}} \right)} \right) \in \; PCG\; \left( {A,B} \right)\Leftrightarrow \left( {x,p,x^{\prime}} \right) \in \; A\; and\; \left( {y,p,y^{\prime}} \right) \in \; B.$$

**Figure 2:**
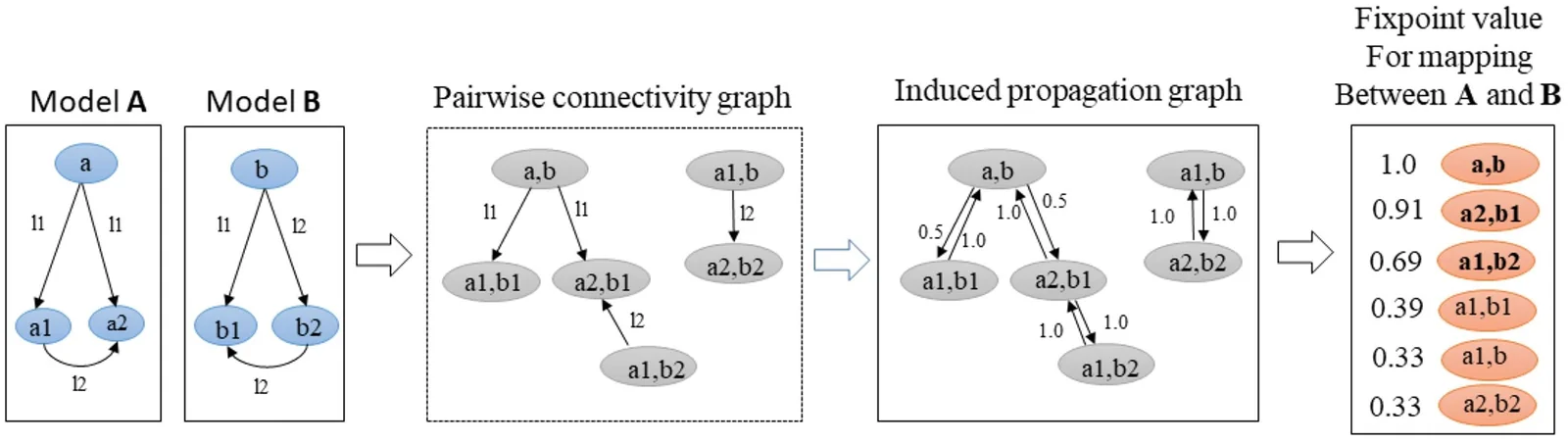
Example illustrating the similarity flooding algorithm.

The induced propagation graph extends the PCG by adding reverse edges and assigning propagation coefficients (ranging from zero to one) to quantify the flow of similarity. These coefficients can be calculated using different methods (Fig. 2). These methods ensure proportional similarity propagation and bidirectional alignment through reverse edges.

Propagation coefficients are determined based on the number of outgoing edges:

- If only one I2-edge originates from a map pair (*e.g*., (a1, b1)), then propagation coefficient w((a1, b)(a2, b2)) is set to 1.0. This indicates that the similarity between a1 and b fully contributes to the similarity between a2 and b2.
- If multiple l2-edges leave a map pair (*e.g*., (a, b)), then the weight of 1.0 is equally distributed among these edges. For instance, if two l2-edges exist, then coefficients w((a, b), (a1, b1) and w((a, b), (a2, b1)) are set to 0.5.

This weighting system ensures proportional similarity distribution across connections while maintaining consistency in similarity propagation.

##### Fixpoint computation

Fixpoint computation is a key step in the SF algorithm, iteratively refining similarity values between elements in two models represented as directed graphs. This process starts with an initial similarity mapping ${{\rm \sigma }^0}$, where each node pair $\left( {x,y} \right)$ is assigned a preliminary similarity value, typically uniform ${{\rm \sigma }^0}\left( {x,y} \right)=1.0$ if no prior information exists.

At each iteration, the similarity values are updated based on contributions from neighboring nodes in the similarity propagation graph, weighted by propagation coefficients. For example, after the first iteration, the similarity value for $\left( {{a_1},{b_1}} \right)$ is updated as ${{\rm \sigma }^1}\left( {{a_1},{b_1}} \right) = {{\rm \sigma }^0}\left( {{{\rm a}_1},{\rm \; }{{\rm b}_1}} \right) + {{\rm \sigma }^0}\left( {{{\rm a}_{\rm u}},{\rm \; }{{\rm b}_{\rm u}}} \right) \cdot 0.5 = 1.5$, while it becomes ${{\rm \sigma }^1}\left( {{\rm a},{\rm b}} \right) = {{\rm \sigma }^0}\left( {{\rm a},{\rm b}} \right) + {{\rm \sigma }^0}\left( {{{\rm a}_1},{\rm \; }{{\rm b}_1}} \right) \cdot 1.0 + {\rm \; }{{\rm \sigma }^0}\left( {{a_2},\; {b_1}} \right) \cdot 1.0 = {\rm \; }3.0$ for $\left( {{\rm a},{\rm b}} \right)$. All values are normalized by dividing by the maximum similarity value in the current iteration to maintain consistency across iterations. For instance, after normalization, ${{\rm \sigma }^1}\left( {a,b} \right) = 1.0$, and ${{\rm \sigma }^1}\left( {{{\rm a}_1},{\rm \; }{{\rm b}_1}} \right) = {{1.5}\over{3.0}} = 0.5$. This normalization step ensures that similarity values remain proportional and comparable across iterations.

The updated similarity mapping ${{\rm \sigma }^{\left\{ {{\mathbf{i}} + 1} \right\}}}\left( {x,y} \right)$ for pair $\left( {x,y} \right)$ is typically computed from the previous iteration ${{\rm \sigma }^{\rm i}}\left( {x,y} \right)$ using the following formula:

(2) $$\eqalign{{{\sigma}^{\left\{ {{\mathbf{i}} + 1} \right\}}}\left( {x,y} \right)  =\ &  {{\sigma }^{\mathbf{i}}}\left( {x,y} \right) + \mathop \sum \limits_{\left\{ {\left( {{{\rm a}_{\rm u}},{\rm \; p},{\rm \; x}} \right) \in {\mathbf{A}},{\rm \; }\left( {{{\rm b}_{\rm u}},{\rm \; p},{\rm \; y}} \right) \in {\mathbf{B}}} \right\}} {{\rm \sigma }^{\mathbf{i}}}\left( {{{\rm a}_{\rm u}},{\rm \; }{{\rm b}_{\rm u}}} \right) \cdot {\mathbf{w}}\left( {\left( {{{\rm a}_{\rm u}},{\rm \; }{{\rm b}_{\rm u}}} \right),\left( {{\rm x},{\rm \; y}} \right)} \right) \\ +& \mathop \sum \limits_{\left\{ {\left( {{{\rm a}_{\rm u}},{\rm \; }{\mathbf{p}},{\rm \; x}} \right) \in {\mathbf{A}},{\rm \; }\left( {{{\rm b}_{\rm u}},{\rm \; }{\mathbf{p}},{\rm \; y}} \right) \in {\mathbf{B}}} \right\}} \matrix{ {{{\rm \sigma }^{\mathbf{i}}}} \cr \; \cr } \left( {{{\rm a}_{\rm v}},{\rm \; }{{\rm b}_{\rm v}}} \right) \cdot {\mathbf{w}}\left( {\left( {{{\rm a}_{\rm v}},{\rm \; }{{\rm b}_{\rm v}}} \right),{\rm \; }\left( {{\rm x},{\rm \; y}} \right)} \right).}$$

The computation is iteratively performed until the Euclidean distance between the residual vectors ${\rm \Delta}\left( {{\sigma ^n},\; {\sigma ^{\left\{ {n - 1} \right\}}}} \right)$ falls below a predefined threshold ε, where $n\; > \; 0$. If convergence is not achieved, then the process stops after reaching a set maximum number of iterations.

#### KGE

KGE aims to represent entities and relationships in KGs as low-dimensional vectors, capturing their semantic structure to enhance specific tasks, such as link prediction and classification (Cao et al., 2024). Research shows that KGE methods vary in effectiveness depending on the techniques and tasks, with factors, such as parallelization, influencing training efficiency and embedding quality (Kochsiek & Gemulla, 2021; Rossi et al., 2021). Despite the potential of KGE methods, they face certain challenges, such as scaling to large datasets with limited computational resources (Liu et al., 2024).

In overcoming these challenges, embedding techniques, such as node2vec, graph attention networks (GATs), and GraphSAGE, have been explored to develop a hybrid method aimed at enhancing Arabic SQS. This approach can potentially lead to significant improvements in task performance. We will explain the embedding techniques in detail as follows.

#### Node2Vec

Node2Vec is an algorithmic framework for learning continuous feature representations of nodes in networks. Unlike traditional methods constrained by rigid network structures, Node2Vec introduces a flexible, biased random walk methodology that interpolates between breadth-first search (BFS) and depth-first search (DFS) to effectively explore the local neighborhood of nodes. This framework allows for the discovery of richer node representations by capturing the structural equivalence and homophily of nodes. The algorithm maps nodes to a low-dimensional feature space, maximizing the likelihood of preserving network neighborhood properties, enabling reliable downstream tasks, such as node classification, link prediction, and clustering (Grover & Leskovec, 2016).

The core of Node2Vec lies in its biased random walks, allowing the algorithm to dynamically explore neighborhoods. A random walk of fixed length l is initiated from a source node u, and the probability of transitioning from node v to x at step i is determined by:

(3) $${\rm P}\left( {{{\rm c}_{\rm i}} = x | {{\rm c}_{\left\{ {{\rm i} - 1} \right\}}} = {\rm \; v}} \right) = \left\{ {\matrix{ {\displaystyle{{{\pi _{vx}}} \over z}\; if\; \left( {v,x} \right) \in E,} \cr {0\; {\rm otherwise}} \cr } } \right.$$

where ${\pi _{vx}}$ represents the unnormalized transition probability between nodes v and x, Z is the normalization constant, and E denotes the edge set of the graph.

The transition probabilities in Node2Vec are guided by two critical parameters: return parameter p and in-out parameter q, which influence the likelihood of revisiting a previously visited node or exploring further nodes. The biased transition probability is defined as ${\pi _{vx = }}{{\rm \alpha }_{pq}}\left( {t,x} \right).{{\rm w}_{{\rm vx}}}$, where ${w_{vx}}{\rm \; }$denotes the edge weight, and ${{\rm \alpha }_{pq}}\left( {t,x} \right)$ is computed based on the shortest path distance ${d_{tx}}$ between the previous node t and the candidate node x. The adjustment factor ${{\rm \alpha }_{pq}}\left( {t,x} \right)$ is calculated using the following equation:

(4) $${{\rm \alpha }_{pq}}\left( {t,x} \right)\left\{ {\matrix{ {\displaystyle{1 \over p}\quad if\; {d_{tx}} = 0} \cr {1\; \quad if\; {d_{tx}} = 0} \cr {\displaystyle{1 \over q}\quad if\; {d_{tx}} = 0} \cr } } \right. .$$

**Figure 3:**
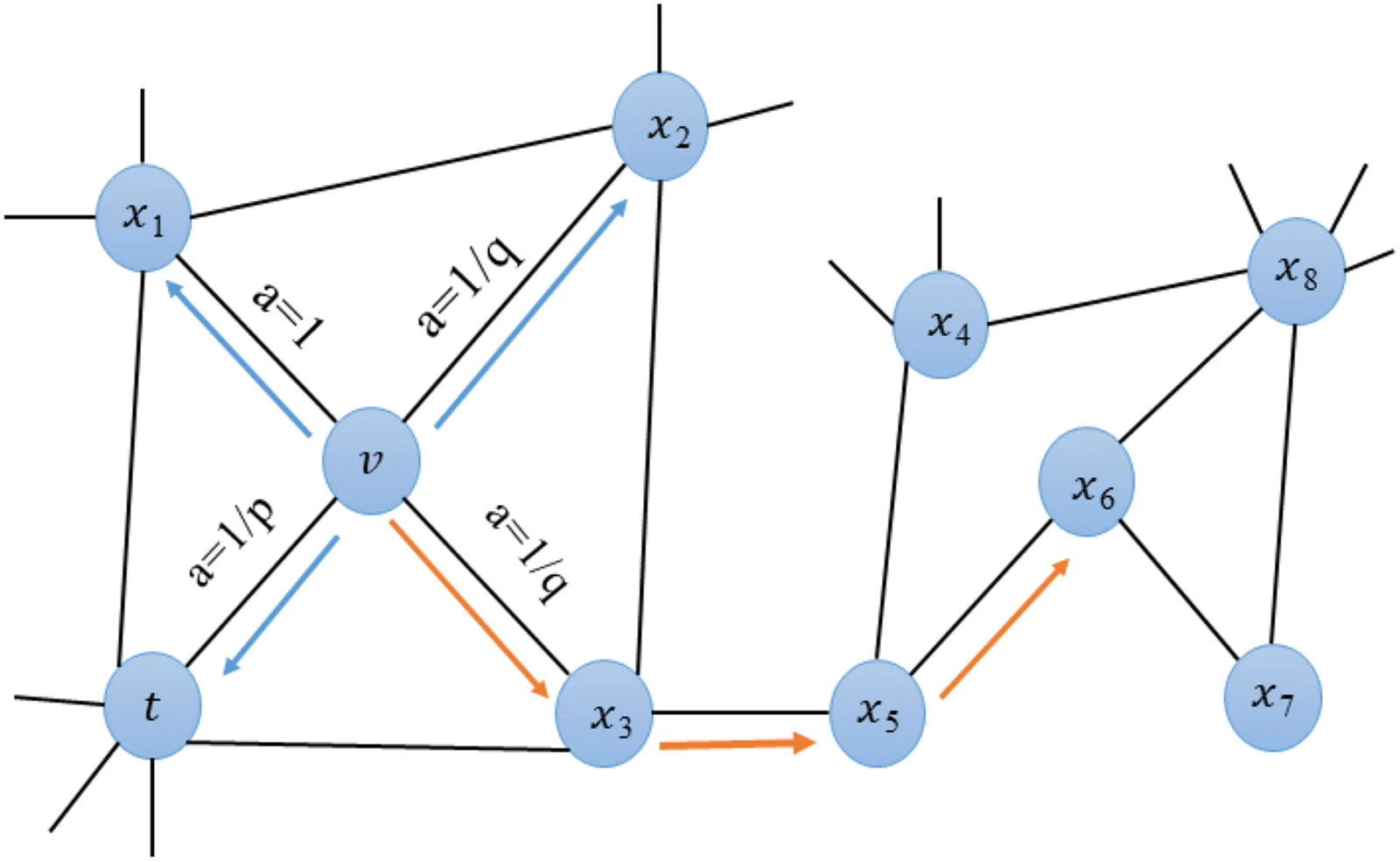
Biased random walk schema incorporating both BFS and DFS.

Node2Vec utilizes a biased random walk schema to balance BFS and DFS strategies (Fig. 3). These parameters, namely, p (return rate) and q (exploration rate), provide fine-grained control over the exploration process. A high $p > 1{\rm \; }$discourages revisiting previous nodes, promoting broader exploration, while a low p<1 encourages localized exploration. Meanwhile, a high q>1 biases the walk toward “inward” nodes closer to the source, resembling BFS-like exploration (blue arrows in the figure), whereas a low q<1 favors “outward” nodes further away, resembling DFS-like exploration (red arrows). Starting from the source node v, random walks of fixed length $\left( {l = 4} \right)$ explore local neighbors $\left( {{x_1},{x_2},{x_3}} \right)$ and deeper, structurally similar nodes $\left( {{x_6}} \right)$, capturing first-order and second-order proximities. This approach ensures meaningful representation learning in complex networks by preserving structural equivalence and homophily (Xu, 2021).

##### GATs

GATs represent an advanced approach for learning from graph-structured data, addressing several limitations of traditional GCNs (Kipf & Welling, 2016). Unlike GCNs, which use fixed weights or spectral methods, GATs employ a self-attention mechanism that dynamically determines the importance of neighboring nodes (Veličković et al., 2017). This mechanism makes GATs highly effective in tasks such as node classification and link prediction, where understanding inter-node relationships is crucial.

To overcome the scalability challenges of GCNs, GATs avoid expensive spectral computations and instead leverage localized attention mechanisms (Veličković et al., 2017). They also handle noisy or irrelevant nodes by assigning adaptive attention weights, reducing the impact of less informative neighbors (Veličković et al., 2017). Furthermore, GATs can model heterogeneous graphs through hierarchical attention mechanisms that capture both node-level and semantic-level relationships (Wang et al., 2019).

The self-attention mechanism computes attention coefficients between connected nodes i and j as follows:

(5) $${{\rm a}_{{\rm ij}}} = \displaystyle{{\exp \left( {{\rm LeakyReLU}\left( {{{\rm a}^{\rm \top }}{\rm \; }\left[ {{\rm W}{{\rm h}_{{\rm i}}}\parallel {\rm W}{{\rm h}_{{\rm j}}}} \right]} \right)} \right)} \over {\mathop \sum \nolimits_{{\rm k} \in {{\rm N}_{\rm i}}} exp\left( {{\rm LeakyReLU}\left( {{{\rm a}^{\rm \top }}\left[ {{\rm W}{{\rm h}_{{\rm i}}}\parallel {\rm W}{{\rm h}_{{\rm k}}}} \right]} \right)} \right)}},$$

Here, ${{\rm h}_{\rm i}}$ and ${{\rm h}_{\rm j}}$ are feature vectors of nodes i and j; W is a learnable weight matrix; a is the attention vector; and ∥ denotes vector concatenation.

The attention coefficients are normalized using the softmax function, allowing the model to focus on the most relevant neighbors during aggregation.

By employing multi-head attention, GATs improve stability and expressiveness, capturing richer relational dependencies and enhancing generalization across graph structures.

##### Graph sample and aggregate (GraphSAGE)

GraphSAGE is a scalable and efficient framework for learning node embeddings in large-scale graph data. This mechanism uses a sampling-based approach to aggregate information from a node’s local neighborhood. This design enables the model to dynamically generate embeddings for unseen nodes or entirely new graphs, making it suitable for real-world applications that involve dynamic and massive graphs (Hamilton, Ying & Leskovec, 2017).

The key idea behind GraphSAGE lies in its ability to efficiently sample and aggregate neighbor information. GraphSAGE samples a fixed number of neighbors for each node instead of relying on all neighbors, which can be computationally expensive. Thereafter, GraphSAGE applies an aggregation function to combine the sampled neighbors’ features. Aggregators, such as mean, LSTM-based, or pooling methods, capture the local and global patterns in the graph. This approach allows GraphSAGE to maintain efficiency without sacrificing representational power.

A critical component of GraphSAGE is its update mechanism, represented by the following equation:

(6) $$h_v^{\left( k \right)} = {\rm \sigma }\left( {{{\rm W}^{\left( {\rm k} \right)}}.AGGREGAT{E^{\left( k \right)}}\left( {\left\{ {{\rm h}_{\rm u}^{\left( {{\rm k} - 1} \right)}:\forall {\rm \; u} \in {\rm N}\left( {\rm v} \right)} \right\}} \right)} \right),$$

where $h_v^{\left( k \right)}$ is the embedding of node v at layer k, and $AGGREGAT{E^{\left( k \right)}}$ is a function that combines features from the node’s neighbors ${\rm N}\left( {\rm v} \right)$. This equation enables GraphSAGE to iteratively refine node embeddings by learning from neighbor information at each layer, making it highly flexible and adaptable to new data.

### Architecture of a hybrid model for identifying Arabic SQS

**Figure 4:**
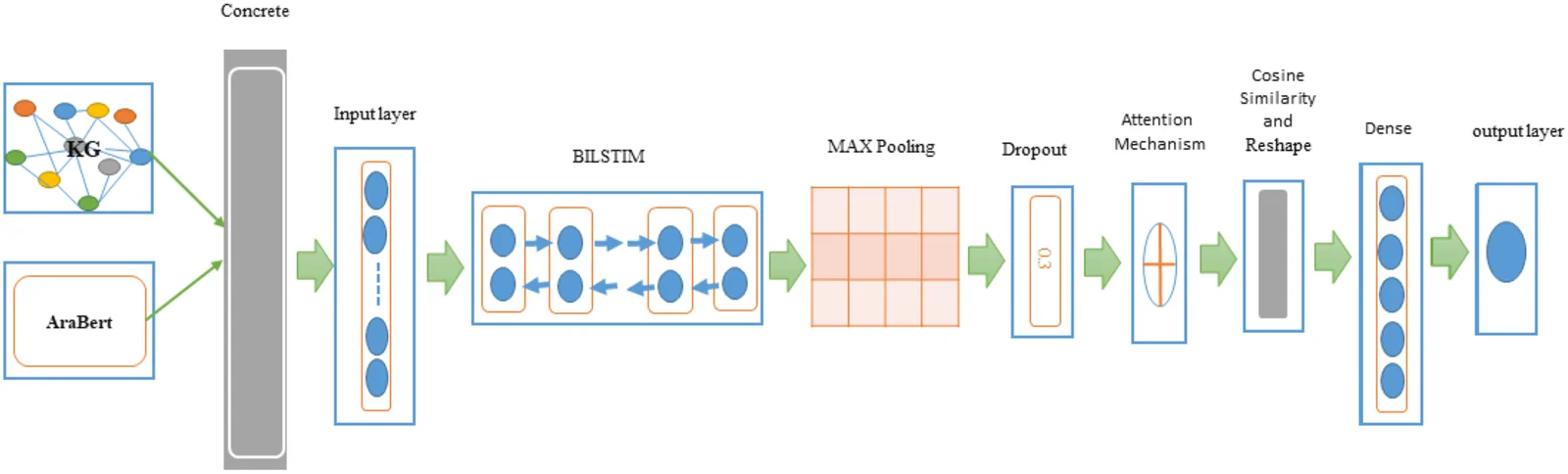
Hybrid model architecture.

This section outlines the main steps of the hybrid model for identifying Arabic SQS, including pre-processing, data augmentation, question representation, and the BiLSTM model. Figure 4 illustrates the question representation and embeddings, along with the BiLSTM model.

#### Pre-processing

Several Arabic preprocessing steps were applied to ensure data consistency and reduce noise: tokenization, stop word removal, text and punctuation cleaning, and orthographic normalization (*e.g*., unifying “إ“, ”أ“, ”آ” to “ا”, and “ة” to “ه”). Segmentation and stemming were performed using the Farasa tool (Abdelali et al., 2016), a widely adopted Arabic NLP toolkit, to obtain standardized root forms. These steps collectively enhanced the dataset’s linguistic quality and improved model input representation.

#### Data augmentation

Deep learning techniques often require large datasets containing both duplicate and non-duplicate question pairs (Hamza et al., 2022), which are difficult to obtain, particularly for Arabic. Data augmentation provides an effective means to overcome this limitation by increasing data diversity and enhancing model generalization (Pellicer, Ferreira & Costa, 2023). Originally developed in computer vision, augmentation has been successfully applied to NLP tasks to enrich training data and mitigate overfitting.

In this study, we employed augmentation strategies based on symmetry, reflexivity, and transitivity relations between question pairs (Shakeel, Karim & Khan, 2020). These relations enable the generation of additional examples by exploiting logical dependencies within the dataset. Specifically, symmetry ensures that if question A is a duplicate of B, then B is also a duplicate of A; reflexivity assumes each question is a duplicate of itself; and transitivity infers that if A is a duplicate of B, and B of C, then A is also a duplicate of C. Conversely, inverse rules were applied to create balanced non-duplicate pairs. This approach effectively expanded the dataset while preserving semantic consistency and improving training stability.

#### Word embedding/question representation

Recent work has demonstrated that improved word representations can be achieved by concatenating different types of embedding (Wang et al., 2021b). Motivated by these findings, our work leverages the strengths of combining embedding from KG-based representations with contextual embedding derived from AraBERT (Antoun, Baly & Hajj, 2020). We aim to enrich the semantic representation of Arabic questions by integrating these complementary representations. KGE captures relational and structural information between words, while AraBERT embedding provide deep contextual understanding. This concatenation approach is suggested to enhance the model’s ability to accurately evaluate the semantic similarity of question pairs.

##### KGE

Building upon the alignment of KGs using the SF algorithm, this study evaluates three distinct KGE techniques: Node2Vec, GATs, and GraphSAGE. These techniques are tested on the Mawdoo3 dataset in the context of the Arabic SQS task. This study aims to identify the most suitable method for capturing the intricate nuances of Arabic SQS by evaluating their performance in terms of accuracy and effectiveness, ultimately contributing to enhanced hybrid model performance.

CatBoost (Prokhorenkova et al., 2018) is a gradient boosting algorithm optimized for handling categorical features while preventing target leakage through ordered boosting. It employs oblivious decision trees to enhance efficiency and reduce overfitting, and processes categorical variables using ordered target statistics to minimize distribution shifts. By dynamically combining categorical features, CatBoost captures complex interactions and often performs competitively compared with models such as XGBoost and LightGBM, offering balanced accuracy and computational efficiency on high-cardinality datasets.

Following the evaluation of KGE techniques, we will compare them with AraVec 2.0 (Soliman, Eissa & El-Beltagy, 2017), an Arabic word embedding model. AraVec 2.0 provides several pre-trained models, including a 300D Wikipedia-Skipgram model. This comparison aims to assess the performance of KGE techniques relative to AraVec 2.0, providing additional insights into their suitability for SQS tasks.

##### AraBERT

Transformers (Wolf et al., 2020) are neural network architectures designed for sequence transduction tasks, such as machine translation, using stacked encoder–decoder layers with self-attention mechanisms. Building on this framework, BERT (Bidirectional Encoder Representations from Transformers) (Devlin et al., 2018) provides bidirectional contextual embeddings through pre-training tasks, including masked language modeling and next sentence prediction. BERT can be fine-tuned for diverse NLP tasks, such as question answering and text classification, and has demonstrated superior performance over previous models. In this study, AraBERT, a pre-trained Arabic version of BERT, was employed to extract contextualized feature representations from Arabic text, effectively capturing semantic and syntactic relationships.

#### BiLSTM model

BiLSTM is a deep learning architecture designed for sequential data, capable of capturing dependencies from both past and future contexts (Graves, Mohamed & Hinton, 2013). This bidirectional structure enables a more comprehensive understanding of contextual semantics, which is particularly valuable for short Arabic texts that often suffer from sparse contextual cues (Pei, 2022). The choice of BiLSTM over CNNs and Transformer-based architectures was driven by theoretical and empirical insights. While CNNs perform well in general text classification by extracting local n-gram features, they often struggle to preserve sequential order and long-range dependencies, which are crucial in semantic similarity tasks (Alian, Himdi & Shaalan, 2025; Sabbeh & Fasihuddin, 2023). In contrast, BiLSTM is well-suited for modeling bidirectional dependencies, capturing the nuanced semantic relationships between question pairs.

Moreover, since BERT and AraBERT were already employed for embedding extraction, adding a Transformer-based decoder would introduce unnecessary computational overhead with minimal performance gain (Alosaimi et al., 2024). In this work, AraBERT provides rich contextual embeddings for each question, while a BiLSTM downstream layer models bidirectional sequential dependencies between question pairs. This design leverages AraBERT’s contextual representations in a lightweight architecture. Studies have shown that combining pre-trained contextual encoders with BiLSTM enhances both accuracy and robustness, particularly for low-resource languages such as Arabic, characterized by complex syntax and morphology (Al-Bataineh et al., 2019; Boudaa, Boudaa & Haddadi, 2024a). The proposed architecture consists of seven main components that jointly capture both structured and contextual semantics, as summarized in Table 1.

**Table 1:** Components of the proposed hybrid BiLSTM-based architecture.

Component	Description
Input layer	Encodes tokenized Arabic text sequences into dense vectors using a hybrid embedding matrix combining KGE and AraBERT contextual features.
BiLSTM layer	Captures bidirectional dependencies by processing embeddings in forward and backward directions, producing contextualized representations of question semantics.
Pooling layer	Applies 1D max-pooling to reduce sequence dimensionality while retaining the most salient contextual features.
Dropout layer	Randomly deactivates 30% of neurons during training to prevent overfitting and improve model generalization.
Attention mechanism	Computes weighted importance over BiLSTM outputs, emphasizing contextually relevant tokens and enhancing long-range semantic dependencies.
Cosine similarity and reshape	Calculates cosine similarity between encoded question pairs and reshapes outputs for subsequent dense-layer processing.
Dense and output layer	Applies a sigmoid-activated dense neuron to produce a final scalar probability representing the semantic similarity between input question pairs.

## Results and Discussion

This section provides a detailed explanation and presentation of all the essential information regarding our experiments, including the datasets used, experimental setup, and discussion of results.

### Dataset

#### Arabic KG alignment resources

This study employs two key resources for KG alignment: Wikidata and LughaNet. Wikidata (https://www.wikidata.org/wiki/Wikidata:Main_Page), an open and multilingual knowledge base maintained by the Wikimedia Foundation, provides structured data accessible *via* both a user interface and the SPARQL query service. The dataset was refined by extracting Arabic entries from a multilingual JSON dump and excluding non-entity pages (*e.g*., “Wikipedia disambiguation page”). Relationships relevant to LughaNet were selected, including Hypernym (subclass of), Instance of, Different from, Said to be the same as, Ordinal number, Alternative name, and Synonym. For illustration, Table 2 presents the entity “lecturer” (Q9379869) and its core relationships. Note that an entity may appear under multiple relation types (*e.g*., both Hypernym and Synonym), as Wikidata allows semantically overlapping links depending on context.

**Table 2:** Semantic relations of the entity “Lecturer” in Wikidata. Data were extracted from the Wikidata JSON dump (Arabic entries), extraction date: June 2024. Non-entity pages were excluded.

(ID)	Label (Arabic/English)	Relations type	ID	Relation label (Arabic/English)
Q9379869	محاضر/lecturer	Instance of	Q28640	مهنه/profession
		Instance of	Q4164871	منصب/position
		Hypernym	Q12859263	خطيب/orator
		Synonym	Q12859263	خطيب/orator

LughaNet, developed at Ferdowsi University of Mashhad, is an extended Arabic WordNet comprising 85,991 synsets, exceeding the original Arabic WordNet’s 11,296 synsets (Cavalli-Sforza et al., 2013). Constructed through a five-stage process using bilingual dictionaries, machine translation, and NLP models (Skip-gram with AraVec 2.0 and BERT), it achieved 64.23% term coverage and an F1-score of 66.61% in Arabic SQS tasks—outperforming Arabic WordNet’s 56.62%. Similar to English WordNet (Fellbaum, 1998), LughaNet organizes words into synsets linked through semantic relations, such as hypernymy, hyponymy, meronymy, and antonymy.

Integrating Wikidata and LughaNet enhances Arabic semantic representation by combining Wikidata’s structured interlinked data with LughaNet’s rich linguistic relations, providing a strong foundation for Arabic KG alignment and improved results in SQS tasks.

#### SQS dataset

We utilized the dataset introduced by Mawdoo3 to predict and evaluate SQS (Seelawi et al., 2019). Each question pair in the dataset is annotated as either semantically similar (label = 1) or not semantically similar (label = 0). The dataset contains a total of 15,712 question pairs, divided into 11,997 for training and 3,715 for testing. The training set includes 6,600 negative (label 0) and 5,397 positive (label 1) pairs, whereas the test set comprises 1,997 negative and 1,718 positive pairs, maintaining a nearly balanced distribution. Data augmentation techniques—based on symmetric, reflexive, and transitive relations—were exclusively applied to the training data to enhance model generalization. Table 3 presents sample entries from the dataset.

**Table 3:** Examples from the Mawdoo3 Q2Q dataset.

Question 1	Question 2	Similarity status
“في أي مدينة يقع متحف الرازي؟” (“In which city is Al-Razi’s museum located?”)	“أين ولد الرازي؟” (“Where was Al-Razi born?”)	No
“أين بدأت الشيوعية؟” (“Where did communism begin?”)	“في أي دولة ظهرت الشيوعية؟” (“In which country did communism appear?”)	Yes

The Mawdoo3 dataset was selected as the primary benchmark because, to the best of our knowledge, it is the only large-scale, publicly available dataset for Arabic SQS. It covers diverse domains with rich linguistic and semantic variation, making it suitable for evaluating Arabic semantic models. Although other Arabic datasets exist, they mainly target paraphrase or entailment tasks rather than question–question similarity.

### Experimental setup

We conducted experiments using Google Colaboratory (https://colab.research.google.com), a local laptop, and the Ferdowsi Cloud (https://ferdowsi.cloud/) computing platform. Google Colaboratory was utilized for tasks that require graphical processing units (GPUs), leveraging its free GPU resources for computationally intensive processes. In other experiments, we utilized a local machine equipped with an AMD Ryzen 7 5800H processor, integrated Radeon Graphics, and 32 GB of RAM. Additionally, given the computational complexity of the GAT model with four attention heads, we implemented it on Ferdowsi Cloud, which provides a Nvidia Tesla A100 GPU, 32 CPU cores, 64 GB of RAM, 200 GB of disk space, and 100 Mb/s shared bandwidth.

We utilized Python libraries, such as NetworkX (https://networkx.org/), Gensim (Řehůřek & Sojka, 2011), Transformers (Wolf et al., 2020), and Node2Vec (Grover & Leskovec, 2016), for KG alignment and KGE experiments. During the SQS experiments, we relied on libraries, including TensorFlow 2.0 (Abadi et al., 2016), Keras (https://keras.io/), PyTorch (Paszke et al., 2019), and Scikit-learn (Pedregosa et al., 2011). These environments and tools were utilized to implement, train, and evaluate our models for KG alignment and SQS tasks.

The KG alignment process utilizes SF to align nodes across graphs. The parameters for this process are summarized in Table 4. The batch_size parameter controls the number of elements processed per batch, ensuring efficient memory usage. The iteration parameter defines the number of similarity propagation cycles to refine similarity scores. The decay_factor adjusts the influence of distant relationships during propagation, while the threshold sets the minimum similarity score required for nodes to be considered a match.

**Table 4:** Similarity flooding parameters.

Parameter	Value
Batch_size	8,000
Iterations	2
Decay_factor	0.85
Threshold	0.45

For reproducibility, the SF process was implemented following the propagation-based schema described in Melnik, Garcia-Molina & Rahm (2002). The initial similarity mapping (σ⁰) was initialized using the cosine similarity between relation vectors extracted from the LughaNet and Wikidata graphs. During each iteration, similarity values were propagated along structurally corresponding edges, weighted by a decay factor (λ = 0.85) to gradually reduce the influence of distant nodes. The process was executed for a maximum of two iterations, which empirically achieved stable convergence across all batches.

The convergence threshold (Δ) was empirically set to 0.001, following a prior study on SF (Melnik, Garcia-Molina & Rahm, 2002), where this value effectively balances convergence stability and computational efficiency. In practice, the process stabilized within two iterations, achieving convergence below this threshold. Finally, entity pairs with a normalized similarity score exceeding the matching threshold (τ = 0.45) were retained as aligned pairs.

After alignment, various graph embedding models were applied to generate node representations for the aligned KGs (Tables 5, 6, and 7). The first model, Node2Vec (Table 5), generates 64-dimensional embeddings by running 200 random walks of 30 steps per node. The model uses a Skip-gram framework with a context window of size 10 and trains in batches of four nodes for computational efficiency. The return and in–out parameters were set to p = 1 and q = 1, respectively, to perform unbiased random walks between breadth-first and depth-first strategies.

**Table 5:** Node2vec model parameters.

Parameter	Value
Dimensions	100
Walk_length	30
Num_walks	200
Workers	4
Window	10
Min_count	1
Batch_words	4
p	1
q	1

**Table 6:** GAT model parameters.

Parameter	Value
In_channels	300
Hidden_channels	128
Out_channels	300
Heads	4
Concat	True (default) for conv1, False for conv2

**Table 7:** GraphSAGE parameters.

Parameter	Value
In_channels	300
Hidden_channels	128
Out_channels	300

The second model, GAT (Graph Attention Network), processes node features through attention mechanisms (Table 6). It receives 300-dimensional input features and maps them to 128-dimensional intermediate representations across four attention heads. The final layer outputs 300-dimensional embeddings, concatenating multi-head outputs in the first layer and averaging them in the second (concat = False).

Similarly, the GraphSAGE model (Table 7) projects 300-dimensional input features into a 128-dimensional hidden layer and produces 300-dimensional embeddings at the output.

Hyperparameters for all models were empirically optimized through extensive experiments to balance accuracy and generalization. Techniques such as dropout and early stopping (patience = 5) were applied to prevent overfitting, with training stopping automatically at epoch 10. During training, each question pair was encoded using AraBERT to obtain contextual embeddings, concatenated with Node2Vec-based KGE, and passed through a BiLSTM layer with an attention mechanism. The final sigmoid layer produced similarity probabilities between question pairs. Training employed a cosine decay learning rate schedule starting at 0.001 with a minimum factor (α = 0.1). The decayed rate was passed to the Adam optimizer (Kingma, 2014), ensuring stable convergence and efficient training performance. The best-performing hyperparameters are summarized in Table 8.

**Table 8:** Hybrid model parameters.

Parameter	Value
Embedding dimensions	KG: 100 + BERT: 768 = 868
LSTM units	128
MaxPooling1D pool size	2
Dropout rate	0.3
Learning rate	Initial: 0.001, decayed (decay_steps: epochs × batch size, alpha: 0.1)
Optimizer	Adam
Batch size	32
Epochs	17
Early stopping patience	5
Loss function	Binary crossentropy
Final layer activation	Sigmoid

### KG alignment and embedding results

The alignment process successfully combines nodes and edges from the LughaNet and Wikidata KGs, resulting in a unified structure that enhances the representation of semantic relationships. The aligned graph comprises 600,298 nodes, 5,075,661 edges, and 48,038 link predicates as shown in Table 9. This integration enables fine-grained semantic connectivity, supporting the development of knowledge-driven applications. Moreover, representation learning—well known for its capacity for feature extraction—is widely applied in the field of NLP.

**Table 9:** Statistics on the alignment of knowledge graphs.

Number of nodes	Number of edges	Number of link predicates
600,298	5,075,661	48,038

In terms of performance, the proposed method achieved an accuracy of 84%, based on a sample of 100 LughaNet entries and their corresponding entities in Wikidata, as evaluated by experts. This performance is comparable to previous unsupervised EA approaches, such as the Similarity Flooding algorithm, which achieved 91% accuracy (Marshall, Chen & Madhusudan, 2006).

**Figure 5:**
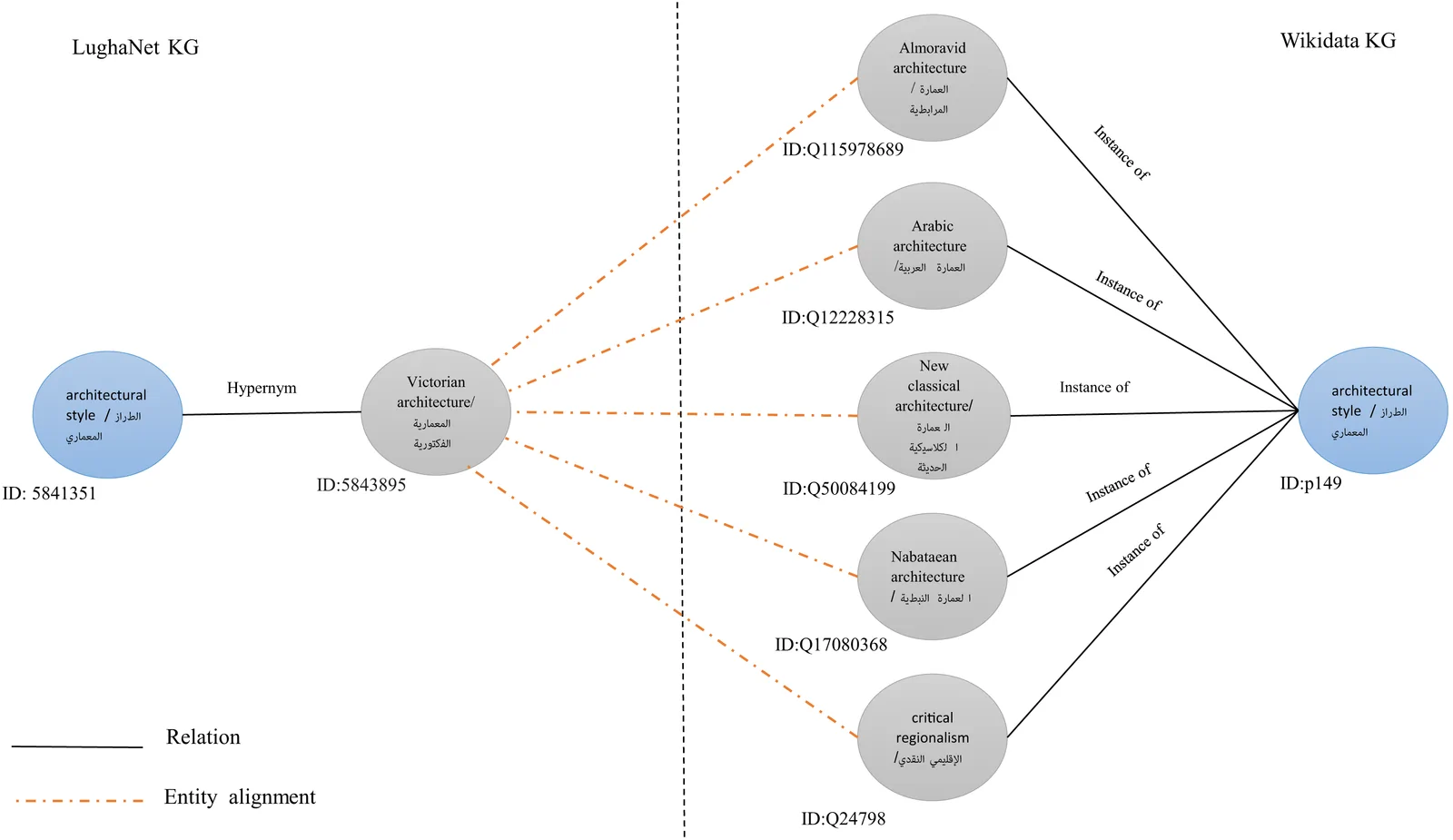
Example of alignment between LughaNet and Wikidata KG.

The alignment process demonstrates the framework’s ability to link semantically similar concepts across KGs. The concept of *Victorian Architecture* (al-mi‘mariyyah al-fikturiyyah, المعمارية الفكتورية) from LughaNet, classified as a hypernym of *Architectural Style* (al-tiraz al-mi‘mari, الطراز المعماري), was successfully aligned with multiple related architectural styles in Wikidata (Fig. 5). These include *Almoravid Architecture* (al-‘imarah al-murabitiyyah, العمارة المرابطية), *Arabic Architecture* (al-‘imarah al-‘arabiyyah, العمارة العربية), *New Classical Architecture* (al-‘imarah al-klasikiyyah al-hadithah, العمارة الكلاسيكية الحديثة), *Nabataean Architecture* (al-‘imarah al-nabatiyyah, العمارة النبطية), and *Critical Regionalism* (al-iqlimi al-naqdi, الإقليمي النقدي), each identified as an instance of *Architectural Style*. This alignment demonstrates the framework’s capability in capturing semantic relationships across KGs.

We utilized CatBoost with the following parameters to evaluate KGE techniques: 400 iterations, a learning rate of 0.1, a depth of 12, and verbose set to 0. Following the evaluation of KGE techniques, we compared the selected KGE method with AraVec 2.0, an Arabic word embedding model that provides pre-trained representations, such as the Wikipedia-Skipgram model with 300 dimensions. The evaluation results highlight the performance metrics (accuracy and F1-score) for each model, as presented in Table 10.

**Table 10:** Results of the evaluation of KGE models.

KGE with CatBoost algorithm	Embedding dimension	Accuracy	F1-score
Node2vec	100	88.15	86.88
GATs	300	86.24	84.72
GraphSAGE	300	85.64	83.75
Ara2vec_wikipedia_sg_300	300	84.68	83.02

The evaluation highlights that Node2Vec stands out despite its smaller embedding dimension (100), which was deliberately chosen to balance computational complexity and resource demands. This trade-off was necessary given the large scale of the aligned knowledge graph, which requires efficient resource management. Despite the reduced embedding size, Node2Vec outperformed other models that employed higher-dimensional embeddings (300), demonstrating its strong capability to encode relational structures effectively.

Furthermore, aligning the KGs significantly enhanced the performance of all embedding models. Node2Vec, GATs, and GraphSAGE each outperformed AraVec 2.0, which relies solely on the Wikipedia-Skipgram model with 300 dimensions. This result underscores the value of KG alignment in improving embedding quality.

Based on the evaluation results, Node2Vec was selected for the hybrid model addressing the SQS task due to its superior overall performance. Using a 100-dimensional embedding, Node2Vec achieved 88.15% accuracy and 86.88% F1-score, outperforming GATs and GraphSAGE despite their use of larger embeddings. Its biased random-walk strategy enables it to capture both local and global relational structures efficiently, making it particularly suitable for large-scale KGs such as the aligned LughaNet–Wikidata KG.

### SQS result

In this section, we present the results of the evaluation of the proposed hybrid model for SQS. The model’s performance is compared with several existing models, with a detailed discussion on the outcomes and observations.

**Figure 6:**
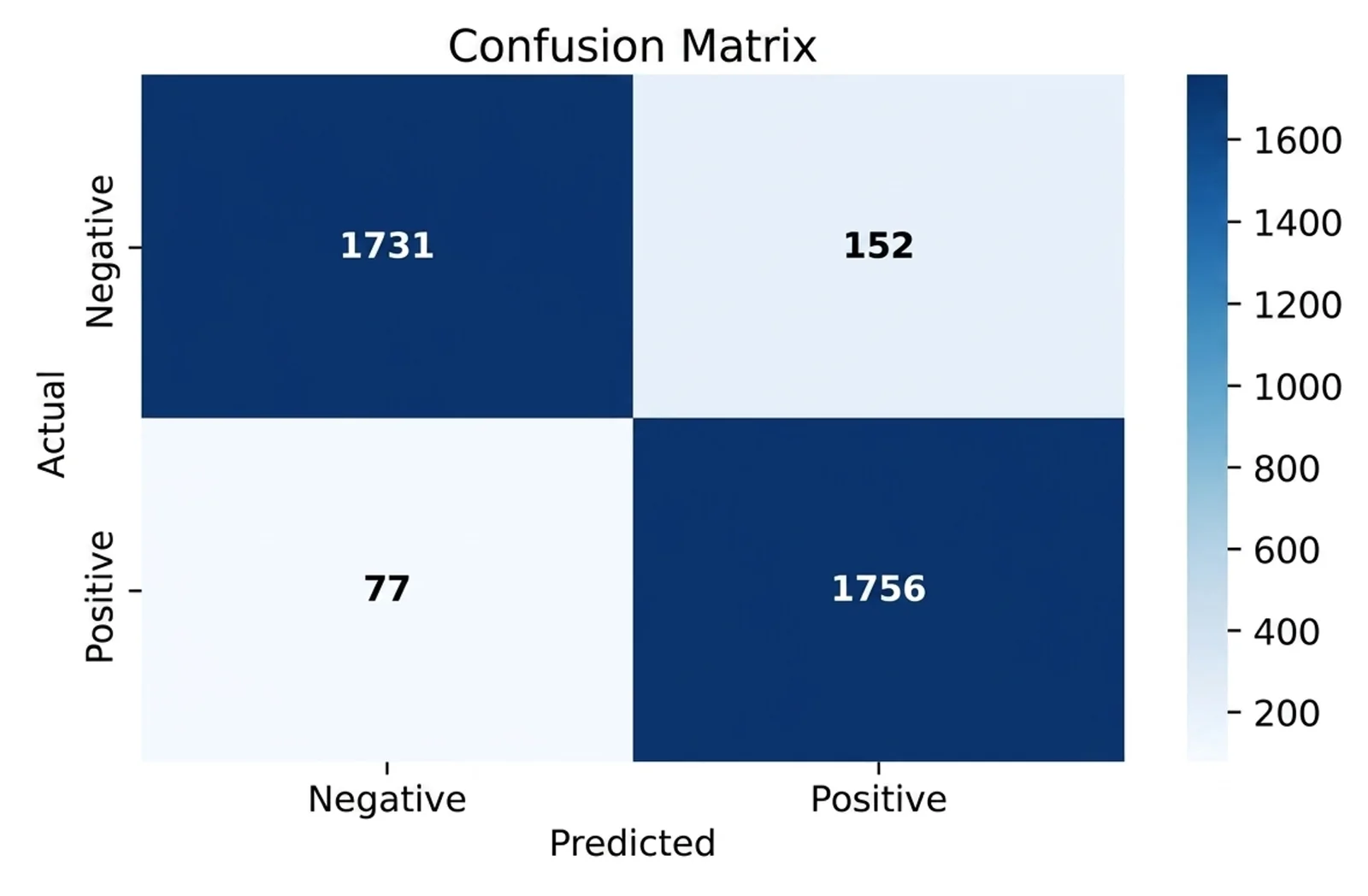
Confusion matrix for the hybrid model.

Table 11 presents the performance metrics of the proposed model, which achieves an accuracy of 93.84%, a recall of 95.80%, a precision of 92.03%, and an F1-score of 93.88%. These metrics reflect the model’s ability to differentiate between semantically similar and dissimilar questions based on the provided dataset. The confusion matrix (Fig. 6) further highlights this aspect, showing 1,731 true negatives, 1,756 true positives, 152 false positives, and 77 false negatives. Although the model performs well overall, the presence of false positives and false negatives suggests that further refinement is needed, especially in differentiating between subtle semantic similarities.

**Table 11:** Performance metrics of the hybrid model.

Accuracy	Recall	Precision	F1-score
93.84%	95.80%	92.03%	93.88%

To assess the contribution of each component, ablation experiments were conducted by selectively removing one module at a time as presented in Table 12. The results show that combining BiLSTM with either AraBERT or the KG yields moderate performance improvements, achieving F1-scores of 92.83% and 92.74%, respectively. However, the full hybrid model that integrates all three components achieves the highest F1-score (93.88%), indicating the complementary roles of contextual embeddings (AraBERT), structured semantic knowledge graphs (KGs), and sequential modeling (BiLSTM).

**Table 12:** Comparison of F1-scores for different model configurations.

Model	F1-score
Bisltm + KG	92.74%
Bilstm + arabert	92.83%
Proposed hybrid model	93.88%

**Figure 7:**
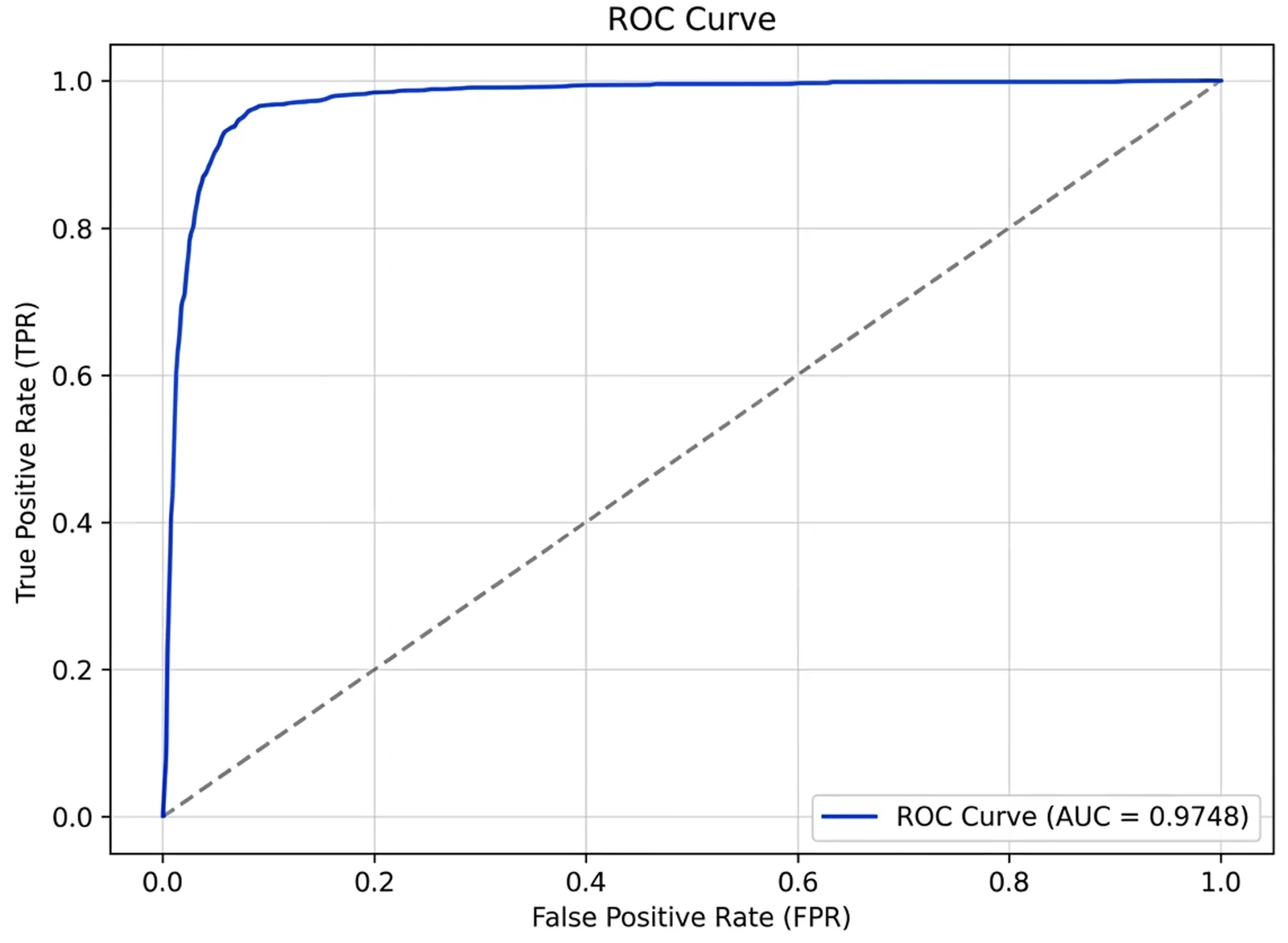
ROC curve for the hybrid model.

The performance of the proposed model was further evaluated using the receiver operating characteristic curve, which is a standard metric for assessing the trade-off between the True Positive Rate (TPR) and the False Positive Rate (FPR) across different classification thresholds (Zou, O’Malley & Mauri, 2007). The model achieves an area under the curve (AUC) of 97.48%, reflecting its ability to distinguish between semantically similar and dissimilar questions (Fig. 7). The high AUC value suggests that the model efficiently performs in balancing sensitivity and specificity.

In Table 13, the training and testing times of the model were compared across different computing platforms to evaluate computational efficiency. The results indicate that Ferdowsi Cloud provides faster and more efficient performance than Google Colaboratory, reflecting the impact of high-performance infrastructure on deep learning model training and inference efficiency.

**Table 13:** Comparison of hybrid model runtime on different computers.

Computer device	Training time	Testing time	Total time
Ferdowsi cloud	10 min 25 s	3 s	10 min 28 s
Google colaboratory	83 min 6 s	11 s	83 min 16 s

Table 14 presents the results of the proposed hybrid model compared with several state-of-the-art approaches. The table highlights the performance of the BERT-based model by Hammad et al. (2021) and the Bi-Attention BiLSTM model by Hamza et al. (2022), alongside GPT-based models (GPT-3.5, GPT-4) (Abdelali et al., 2024), the KG-Embedding-SVM model by Boudaa, Haddadi & Boudaa (2024b), and FArSS+ by Alkaoud (2025).

**Table 14:** Comparison of the proposed hybrid model with state-of-the-art models.

Ref.	Model name	Performance	
		Accuracy	F1-score
Abdelali et al. (2024)	GPT-3.5 (zero-shot)		81.60%
Boudaa, Haddadi & Boudaa (2024b)	KG-embedding-SVM	78.50%	81.764%
Abdelali et al. (2024)	GPT-4 (zero-shot)		88.10%
Alkaoud (2025)	FArSS+		92.80%
Hammad et al. (2021)	BERT-based		92.99%
Hamza et al. (2022)	BiAttention BiLSTM	93.05%	93.05%
Abdelali et al. (2024)	Few-shot GPT-4 (3-shot)		93.50
Seelawi et al. (2019)	Ensemble of BERT	95.92%	
**Our proposed model**	**(Knowledge graph-AraBert-BiLSTM)**	**93.84%**	**93.88%**

**Note:**

The proposed model is shown in bold.

Although recent GPT models have shown noticeable progress, as reported by Abdelali et al. (2024)—with F1-scores of 81.60% for GPT-3.5, 88.10% for Zero-Shot GPT-4, and 93.50% for Few-Shot GPT-4—our proposed method surpasses the GPT baselines (GPT-3.5 and Zero-Shot GPT-4) and slightly outperforms Few-Shot GPT-4 by 0.33 points. This highlights that while GPT models possess strong generalization capabilities, Arabic NLP still benefits from specialized tools such as KGs.

Previous attempts to use KGs, including Arabic WordNet and Wikidata, have faced limitations (Boudaa, Haddadi & Boudaa, 2024b). For instance, Arabic WordNet, comprising 11,296 synsets, primarily captures synonymy but lacks deeper semantic relations required for SQS tasks. Moreover, traditional learning models like SVM restrict the effective integration of such structured resources, achieving only 78.50% accuracy and 81.76% F1-score in prior studies.

In contrast, our model leverages the newly constructed LughaNet KG, comprising 85,991 synsets and aligned with Wikidata (600,298 nodes and 5,075,661 edges). By integrating rich semantic relations such as hypernyms, hyponyms, and meronyms, this hybrid approach enhances semantic representation and bridges gaps left by traditional methods.

Comparable studies employing embedding-based models, such as FArSS+ (Alkaoud, 2025) with fastText (F1 = 92.80%) and (Hamza et al., 2022) with ELMo-based BiLSTM (F1 = 93.05%), achieved strong results but were surpassed by our approach. Similarly, the BERT-based model by Hammad et al. (2021) reached an F1-score of 92.99%, while our hybrid model, combining AraBERT, BiLSTM, and KGE, achieved 93.88%.

The highest reported accuracy on the Mawdoo3 dataset (95.92%) was achieved by the Inception team in the NSURL 2019 shared task (Seelawi et al., 2019) using an ensemble of 3–6 fine-tuned BERT models with majority voting. In comparison, our single hybrid model—combining knowledge graph embeddings (KGE) with AraBERT and BiLSTM—achieves 93.84% accuracy and a 93.88% F1-score without ensembling. Although the performance does not surpass the reported SOTA, it remains highly competitive, offering comparable accuracy with significantly reduced computational overhead and improved interpretability through explicit KG integration.

To quantitatively assess the contribution of KGE, we compared the performance of the proposed hybrid model (AraBERT + BiLSTM + KG) with a reduced variant excluding the KG component (AraBERT + BiLSTM). The models were evaluated across five independent test partitions, each containing 743 samples as presented in Table 15. The hybrid model consistently outperformed the baseline, achieving mean F1-scores of 94.09% and 91.83%, respectively. A paired t-test yielded *t* = 6.11 and *p* = 0.0036, indicating that the improvement is statistically significant (*p* < 0.05) and therefore unlikely to have occurred by chance. These findings confirm that incorporating structured KG representations significantly enhances semantic understanding and model robustness.

**Table 15:** Performance comparison between hybrid and non-KG models across test partitions.

Data	F1-score hybrid model	F1-score model without KG
Data 1	94.85%	91.45%
Data 2	93.30%	92.13%
Data 3	93.92%	92.02%
Data 4	95.12%	92.52%
Data 5	93.27%	91.05%

#### Error predicates

**Figure 8:**
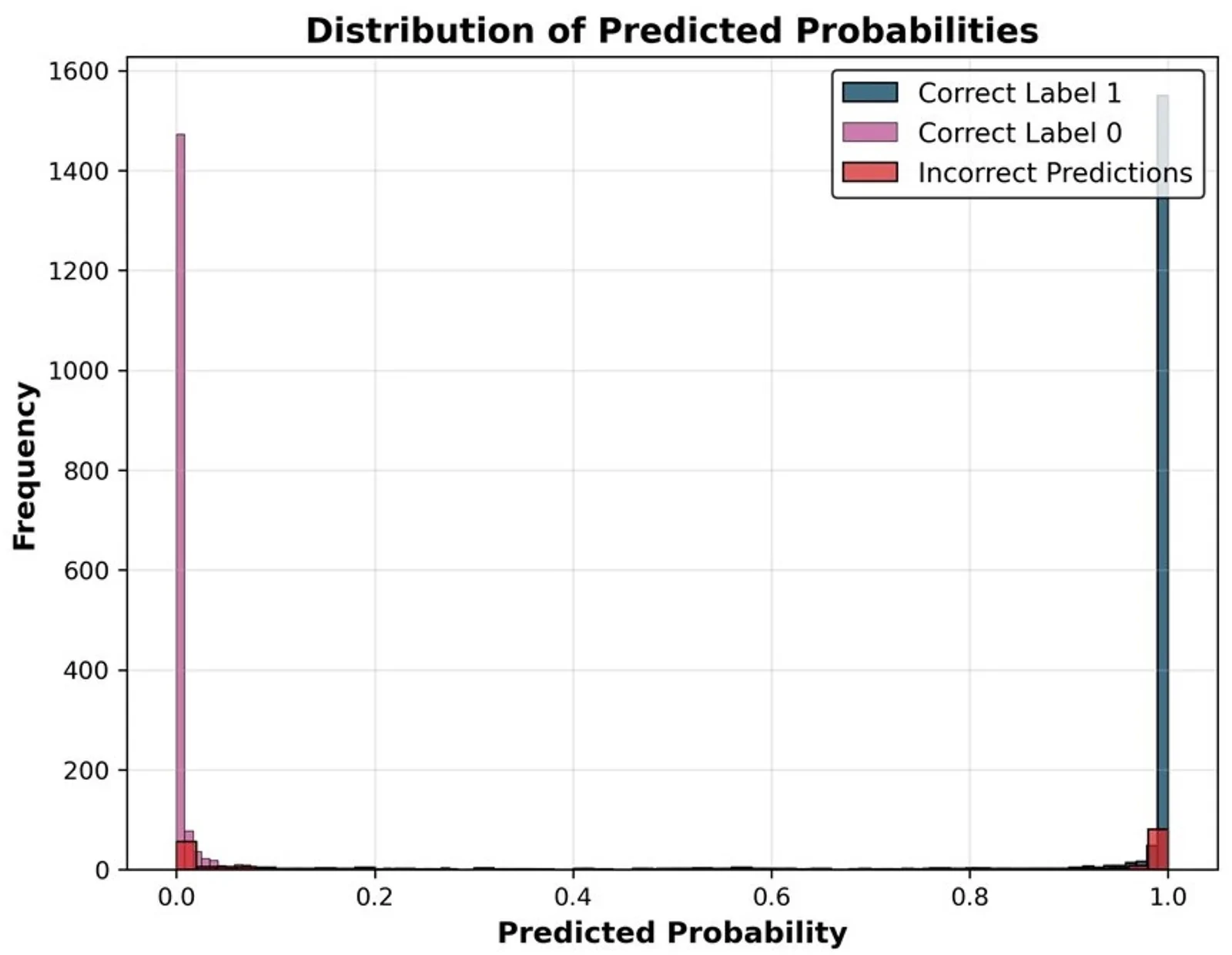
Distribution of predicted probabilities.

To further evaluate the model’s performance and identify its limitations, an error analysis was conducted on misclassified question pairs. Figure 8 illustrates the probability distribution of the model’s predictions for the binary SQS classification task, showing confidence levels for similar (Label 1) and dissimilar (Label 0) question pairs. Correct predictions for both classes cluster near 0.0 and 1.0, whereas misclassifications are scattered across intermediate probabilities, reflecting varying degrees of model uncertainty.

Table 16 summarizes three representative misclassified cases that reveal key sources of error in Arabic question similarity classification.

**Table 16:** Sample of misclassified questions across preprocessing stages (before/after) and translations.

Cases		Question 1	Question 2	True label	Predicted label	Predicted probability
Case 1	Before preprocessing	ما المحافظة الأردنية التي توجد في أقصى شمال شرق الأردن؟	أين تقع محافظة المفرق في الأردن؟	Similar	Not similar	0.00036563
	Translation	Which Jordanian governorate is located in the far northeast of Jordan?	Where is Mafraq Governorate located in Jordan?			
	After preprocessing	ما محافظه اردني اقصي شرق اردن؟	اين وقع محافظه مفرق اردن؟			
	Translation	What easternmost Jordanian governorate?	Where Mafraq Governorate located Jordan?			
Case 2	Before preprocessing	ماهي اعراض الصفار؟	ما هي اعراض الصفراء؟	Not similar	Similar	0.9813592
	Translation	What are the symptoms of pallor/jaundice?	What are the symptoms of Bile Duct?			
	After preprocessing	ما اعراض صفار؟	ما اعراض اصفر؟			
	Translation	What symptom yellow?	What symptoms yellow			
Case 3	Before preprocessing	ما هي طرق الاهتمام بالبشرة الدهنية؟	ما هي طرق الاهتمام بالبشرة؟	Not similar	Similar	0.9988471
	Translation	What are the ways to care for oily skin?	What are the ways to care for the skin?			
	After preprocessing	ما طريق اهتمام بشره دهني؟	ما طريق اهتمام بشره؟			
	Translation	What way care oily skin?	What way care skin?			

*Case 1–Preprocessing Deletion Error:* Preprocessing removed key words such as “توجد” (tūjad, “located”) and “شمال” (shamāl, “north”) from Question 1, altering its meaning. Since Question 2 remained semantically intact, the model misclassified the pair as not similar despite their underlying equivalence.

*Case 2–Stemming Ambiguity:* Stemming errors caused medical terms such as “الصفار” (aṣ-ṣafār, jaundice) and “الصفراء” (aṣ-ṣafrāʾ, bile duct) to be incorrectly reduced to the adjective yellow, leading to a false positive classification.

*Case 3–Modifier Sensitivity:* The model failed to distinguish subtle differences between near-duplicate questions that varied only by modifiers such as “دهني” (duhnī, oily), highlighting its difficulty in capturing fine-grained semantic variations.

These examples emphasize the linguistic and morphological challenges of Arabic question similarity, particularly the need for improved preprocessing, stemming, and semantic disambiguation tools to reduce high-confidence misclassifications. The KG used in this study includes only semantically meaningful words—such as nouns, verbs, adjectives, and adverbs—excluding stop words without standalone meaning. Therefore, stop-word removal and stemming were intentionally applied to ensure alignment between question terms and KG entities (*e.g*., “المعلمين” (al-mu‘allimīn, teachers), aligned to “معلم” (mu‘allim, teacher)). Although these steps may introduce minor semantic loss, they are essential for maintaining lexical consistency and effective KG integration.

## Discussion

The KG alignment and embedding results demonstrate the importance of integrating structural and semantic knowledge to enhance representation quality in Arabic KG. Aligning LughaNet with Wikidata produced a unified, semantically enriched structure that improved relational understanding across domains. Node2Vec outperformed more complex models such as GATs and GraphSAGE, confirming that bias-controlled random walks effectively capture both local and global dependencies in large-scale KGs. The observed gains after alignment indicate that maintaining semantic consistency between nodes strengthens downstream tasks such as SQS. Overall, the hybrid approach achieves a practical balance between accuracy, scalability, and interpretability.

The SQS results further highlight the effectiveness of combining structured and contextual representations in Arabic semantic modeling. The statistically significant improvement obtained by incorporating KGE demonstrates that structured semantic information complements contextual embeddings beyond purely text-based approaches. While the proposed hybrid framework delivers competitive performance compared to ensemble and GPT-based systems, the analysis also reveals challenges in addressing fine-grained linguistic variations and morphological ambiguities inherent in Arabic. These findings emphasize the need for linguistically informed preprocessing pipelines and scalable KG integration strategies to advance Arabic semantic modeling and improve cross-domain generalization.

## Conclusions

This study addresses the critical challenge of SQS in Arabic—a morphologically rich and semantically ambiguous language with limited linguistic resources. To enhance semantic representation, we propose a hybrid framework that integrates KGs, contextual embeddings, and deep learning models. The approach comprises two main components: (1) KG alignment and embedding generation, and (2) hybrid model implementation.

In the first stage, LughaNet and Wikidata were aligned using the SF algorithm to construct a unified KG containing 600,298 entities, 5,075,661 relations, and 48,038 predicates. Among the evaluated embedding methods, Node2Vec achieved the highest F1-score (86.88%), outperforming GraphSAGE, GATs, and the AraVec 2.0 baseline. In the second stage, Node2Vec embeddings were combined with contextual representations from AraBERT and processed through a BiLSTM-attention model for question similarity prediction.

The proposed hybrid model achieved 93.84% accuracy, 93.88% F1-score, and 97.48% AUC on the Mawdoo3 dataset—surpassing GPT-based and traditional machine learning baselines. These results demonstrate the effectiveness of the hybrid model in improving semantic understanding in Arabic NLP.

However, several limitations remain. Preprocessing tools such as stemming and stop-word removal may cause minor semantic loss, while domain-specific terminology—particularly in medical contexts—poses additional challenges. Furthermore, the model’s generalization across Arabic dialects remains untested, and large-scale KG alignment imposes notable computational costs that affect scalability. Future work will extend evaluation and cross-validation using additional Arabic datasets as they become publicly available, further testing the generalization capability and robustness of the proposed hybrid framework.

Overall, this study presents one of the first hybrid KG—embedding approaches for Arabic SQS, offering both competitive performance and interpretability. Future research should aim to develop more robust Arabic text preprocessing methods, construct domain-specific KGs (*e.g*., healthcare, law, and education), and integrate them with general-purpose graphs. Enhancing explainability and interpretability will also be essential for improving trust and transparency in real-world applications.

## Supplemental Information

10.7717/peerj-cs.3988/supp-1 Supplemental Information 1 Semantic Question Similarity Dataset.

10.7717/peerj-cs.3988/supp-2 Supplemental Information 2 Translation codebook for the Semantic Similarity Dataset and Training Sets.

10.7717/peerj-cs.3988/supp-3 Supplemental Information 3 Code.

## Additional Information and Declarations

### Data Availability

The following information was supplied regarding data availability:

The raw data and code are available in the Supplemental Files.

The dataset is available at Kaggle: https://www.kaggle.com/c/nsurl-2019-task8/data.

The implementation code is available at Zenodo: Al_edhari, A., & Kahani, M. (2025). Enhancing Semantic Question Similarity in Arabic Using Hybrid Models. Zenodo. https://doi.org/10.5281/zenodo.15092523.
